# Apoptosis induction in cancer cell lines and anti-inflammatory and anti-pathogenic properties of proteinaceous metabolites secreted from potential probiotic *Enterococcus faecalis* KUMS-T48

**DOI:** 10.1038/s41598-023-34894-2

**Published:** 2023-05-15

**Authors:** Faezeh Salek, Hamid Mirzaei, Jalil Khandaghi, Afshin Javadi, Yousef Nami

**Affiliations:** 1 Department of Food Hygiene, Faculty of Veterinary Medicine, Tabriz Medical Sciences, Islamic Azad University, Tabriz, Iran; 2 Department of Food Biotechnology, Biotechnology Research Center, Tabriz Branch, Islamic Azad University, Tabriz, Iran; 3 Department of Food Science and Technology, Sarab Branch, Islamic Azad University, Sarab, Iran; 4 Department of Food Biotechnology, Branch for Northwest and West Region, Agricultural Biotechnology Research Institute of Iran, Agricultural Research, Education and Extension Organization (AREEO), Tabriz, Iran

**Keywords:** Cell biology, Microbiology

## Abstract

Potential probiotic *Enterococcus faecalis* KUMS-T48, isolated from a kind of Iranian traditional dairy product (Tarkhineh), was assessed for its anti-pathogenic, anti-inflammatory and anti-proliferative properties against HT-29 and AGS cancer cell lines. This strain showed strong effects on *Bacillus subtilis* and *Listeria monocytogenes* and moderate effect on *Yersinia enterocolitica*, while indicated weak effect on *Klebsiella pneumoniae* and *Escherichia coli*. Also, neutralizing the cell-free supernatant and treating it with catalase and proteinase K enzymes reduced the antibacterial effects. Similar to Taxol, the cell-free supernatant of *E. faecalis* KUMS-T48 inhibited the in vitro proliferation of both cancer cells in a dose-dependent manner, but unlike Taxol, they had no activity against normal cell line (FHs-74). Pronase-treatment of the CFS of *E. faecalis* KUMS-T48 abrogated its anti-proliferative capacity, thereby showing the proteinaceous nature of the cell-free supernatant. Further, induction of apoptosis-based cytotoxic mechanism by *E. faecalis* KUMS-T48 cell-free supernatant is related to anti-apoptotic genes ErbB-2 and ErbB-3, which is different from Taxol’s apoptosis induction (intrinsic mitochondria apoptosis pathway). Also, as evidenced by a decline in interleukin 1β inflammation-promoting gene expression and a rise in the anti-inflammatory interleukin-10 gene expression in the HT-29 cell line, probiotic *E. faecalis* KUMS-T48 cell-free supernatant demonstrated a significant anti-inflammatory impact.

## Introduction

Recent discoveries on the benefits of food bacterial populations on human health motivate further interest in bacterial communities and their functions^1,2^. Tarkhineh is a kind of traditional fermented food that is popular in the western provinces of Iran as well as in many different countries such as Armenia, Iraq, Turkey, Greece, and Bulgaria^3^. Iranian Tarkhineh, which is rich in probiotic lactic acid bacteria (LABs), is made up of wheat or barley grouts that is cooked for hours in sour milk, and the pieces of this paste-like mixture are balled and dried after adding local vegetables such as turnips and oregano^4^.

Probiotic strains with health-promoting properties are known as bio-therapeutic agents^5^. Several studies have revealed that the majority of probiotic bacteria belong to the lactic acid bacteria group^6^. LABs exhibit fermentation activities and have been used in food preservation for thousands of years. The LAB groups include the genera such as *Lactobacillus*, *Lactococcus*, *Enterococcus*, *Leuconostoc*, *Oenococcus*, *Streptococcus*, and *Pediococcus*^7,8^.

*Enterococcus* has the third highest number of species in the LAB group following *Lactobacillus* and *Streptococcus* genera^9^. They are catalase-negative, Gram-positive, and non-spore-forming bacteria found in a variety of fermented foods^10^. *E. faecium* and *E. faecalis* are two predominant species among *Enterococcus* species^11^. The application of probiotic *Enterococcus* species has been recently augmented as a consequence of their affirmative effects on human health^12^.

Probiotics, as health-promoting microorganisms, show different therapeutic properties such as anti-pathogenic and cholesterol-lowering activities^13^. However, anti-carcinogenic activity is the most interesting property that has been linked to probiotics^14,15^. In this regard, different studies have also been conducted on the anti-proliferative effects of food-derived enterococci on various cancer cell lines^16–18^. Apoptosis induction and down-regulation of genes involved in cancer cell proliferation are two effective mechanisms of probiotic cell-free supernatant in protective effects against many cancers^19,20^. This study aimed to assess the anti-carcinogenic, anti-inflammatory, and anti-pathogenic activity of probiotic *Enterococcus faecalis* KUMS-T48 isolated from Tarkhineh.

## Material and methods

### Bacterial growth condition

The present study was conducted on *Enterococcus faecalis* KUMS-T48, which was previously isolated from Tarkhineh, and its molecular identification and potential probiotic properties were also evaluated^7^. The de Man Rogosa Sharpe (MRS) medium was used for the activation and growth of this strain.

### Antimicrobial activity

The modified well diffusion agar method previously described by Nami et al.^21^ was used to determine the antagonistic effect of *E. faecalis* KUMS-T48 cell-free supernatant on five indicator pathogens including *Listeria monocytogenes* (PTCC 1163), *Escherichia coli* (PTCC 1276), *Klebsiella pneumoniae* (ATCC 43,816), *Yersinia enterocolitica* (ATCC 2715), and *Bacillus subtilis* (ATCC 19,652). For this, 50 μL of the filtrate (through 0.2 µm filter) of an overnight culture of *E. faecalis* KUMS-T48 in MRS broth at 37 °C was added to 7 mm diameter wells on MRS agar plates (Sigma-Aldrich, USA), which were previously surface cultured with indicator pathogens and incubated overnight at 37 °C. After overnight incubation of plates at 37 °C, the clear zones around of each well were measured and considered as positive antibacterial activity. According to the diameter of the inhibition zone (average of two perpendicular diameters), the anti-pathogenic activity was divided into strong (diameter ≥ 20 mm), moderate (10 mm < diameter < 20 mm), and weak (diameter ≤ 10 mm). In order to determine the primary ingredient involved in the antagonistic properties of the cell-free supernatant, the neutralized form (adjusted to pH 7.2 by adding 1 M NaOH) and treated forms of the cell-free supernatant with catalase and proteinase K enzymes were also subjected to antibacterial tests.

### Anti-proliferative activity

#### Preparation of cell-free supernatant (CFS)

For this purpose, the selected *Enterococcus* strain was cultured on MRS broth and incubated at 37 °C for 24 h. By measuring the bacterial optical density (OD), 1 × 10^6^ CFU mL^−1^ (standard number of viable probiotic cells) was chosen as the ready-to-use cell culture. The active supernatants were separated via centrifugation at 4000× *g* for 30 min at 4 °C of cell cultures. Then, the supernatant was filtered through a 0.22 µm pore-size filter after neutralizing by adding 1 M NaOH^22^.

#### Growth condition of cell lines

The human gastric cancer cell line (AGS), human colon cancer cell line (HT-29), and human normal cell line (FHs-74), which were provided by the Tehran Institute of Pasteur (Tehran, Iran), were employed in the bacteria-secreted metabolite cytotoxicity assays^23^. All cell lines were cultured in 96-well cell culture plates containing Dulbeccos Modified Eagles Medium (Sigma-Aldrich, USA), where the media were supplemented with 100 IU mL^−1^ penicillin, 10 µg mL^−1^ streptomycin, and 10% (v/v) fetal bovine serum at similar incubation conditions for all cells (37 °C, 95% humidity, and 5% CO_2_). All cultured cells were subsequently treated with 12 concentration rates (5–60 µg mL^−1^) of resulted CFS to screen the effective and suitable concentration for continuing the experiments at three-time points (12, 24, and 48 h).

#### Cell viability assessment by MTT test

The MTT (3-(4,5-dimethylthiazol-2-yl)-2,5-diphenyltetrazolium bromide) assay was used to detect viable cells^24^. For this, the cancer cell lines and normal cell lines were plated in the 96-well microplate. Each well was seeded with 1.2 × 10^4^ cells in 150 µl of growth medium. After 24 h post-seeding (40% to 60% confluency), 50 µL (30% (v/v)) of the CFS were administered to each well to achieve a total volume of 200 µL. All treated cells were then incubated for 12, 24, and 48 h. After incubation, 50 µl of MTT solution (5 mg ml^−1^ in PBS) was administered to each microplate well. Then, the plates were again incubated for 4 h in 5% CO_2_ at 37 °C in dark conditions. The formazan crystals created by using MTT-exposed live cells were dissolved by adding 200 µl of dimethyl sulfoxide (DMSO) and 25 µL of Sorenson’s glycine buffer (0.1 M glycine and 0.1 M NaCl, pH 10.5) into each well and were incubated at 37 °C while being gently shaken for 20 min. Adsorption of the dissolved formazan crystals was measured using a μQuant ELISA Reader (Biotek, ELx 800, USA) at 570 nm. The cytotoxic assessments were continued using Taxol-treated groups as a positive control, and untreated cell lines as a negative control. For further comparison, the results were compared with the cell-free supernatant of the reference *E. faecalis* strain (*E. faecalis*, PTCC 1774).

#### Apoptosis assessment by DAPI staining

This test was carried out using the previously described method^25^. Briefly, all treated/untreated cell lines were cultured on sterile cover slip sets at the bottom of each 6-well cell culture plate (cell density 120 × 10^4^/each well). At the end of each time point (12, 24, and 48 h), the cells were fixed by 4% paraformaldehyde (PFA) for 5 min and were permeabilized by 0.1% Triton X-100 for 5 min. Then, 50 µl of diluted 4′,6-diamidino-2-phenyl indole (DAPI) was diluted 1:2000 with its buffer (FOXP3 Perm Buffer, BioLegend, San Diego, USA) and added to each well. After 5 min incubation at room temperature, the cells were washed with sterile PBS (pH 7.2). The stained cells on cover slips were reversely placed on the slides and then were analyzed using a fluorescent microscope (Olympus BX61, Center Valley, PA, USA) equipped with a U-MWU2 fluorescence filter (excitation filter BP 330–385, dichromatic mirror DM 400, and emission filter LP 420).

#### RNA analysis of apoptotic genes

Real-time PCR was used to examine seven apoptotic expression genes, including ErbB-2, ErbB-3, BAX, BCL-2, BCL-XL, CASP-8, and CASP-9 (Table 1). AGS cancer cells were lysed for RNA analysis using TRI Reagent R (Sigma Chemical Co., Poole, UK) according to manufacturer instructions. To accomplish this, 24 h post-treatment or untreated control monolayer cells were lysed, and the air-dried samples were dissolved in DEPC-treated water and tested qualitatively and quantitatively before use in RT-PCR experiments. Moloney-Murine Leukemia Virus (MMLV) reverse transcriptase was used to convert the isolated RNA to cDNA (Bethesda Research Laboratories, Gaithersburg, MD, USA). One µL RNA (1 µg/L) was combined with a master mix [DEPC-treated water, 13 µL, dNTPs (10 µM) 2 µL, MMLV buffer with DTT: 2 µL, random hexamer primer (pdN6; 400 ng/µL) 0.5 µL] and denatured at 95 °C for 5 min for the RT reaction. The sample was then chilled to 4 °C in an ice bath for 5 min. The sample was then incubated with one µL MMLV (200 U/µL) and 0.5 µL RNase (40 U/µL) using the following thermocycling program: 10 min at 25 °C, 42 min at 42 °C, and 5 min at 95 °C. Real-time PCR assays were conducted using the generated cDNA templates.

**Table 1:** Primers used for real time PCR to detect apoptotic and anti-inflammatory genes in cancerous cells.

Primer	Forward and reverse primer	Sequences	Amplicon size	Length	References
Apoptotic and anti-apoptotic primers					
ErbB2	F	5′-TGTGACTGCCTGTCCCTACAA-3′	152	21	
	R	5′-CCAGACCATAGCACACTCGG-3′		20	
ErbB3	F	5′-GACCCAGGTCTACGATGGGAA-3′	99	21	
	R	5′-GTGAGCTGAGTCAAGCGGAG-3′		20	
BCL-XL	F	5′-GAGCTGGTGGTTGACTTTCTC-3′	101	21	
	R	5′-TCCATCTCCGATTCAGTCCCT-3′		21	^10^
BCL-2	F	5′-GGTGGGGTCATGTGTGTGG-3′	130	19	
	R	5′-CGGTTCAGGTACTCAGTCATCC-3′		22	
BAX	F	5′-CCCGAGAGGTCTTTTTCCGAG-3′	155	21	
	R	5′-CCAGCCCATGATGGTTCTGAT-3′		21	
CASP-8	F	5′-GACAGAGCTTCTTCGAGACAC-3′	116	21	
	R	5′-GCTCGGGCATACAGGCAAAT-3′		20	
CASP-9	F	5′-CTCAGACCAGAGATTCGCAAAC-3′	116	22	
	R	5′-GCATTTCCCCTCAAACTCTCAA-3′		22	
Anti-inflammatory primers					
IL-1β	F	5′-GCTTATTACAGTGGCAATGA-3′	129	20	
	R	5′-GTGGTCGGAGATTCGTAG-3′		18	^26^
IL-10	F	5′-TGGAGGACTTTAAGGGTTAC-3′	114	20	
	R	5′-GATGTCTGGGTCTTGGTT-3′		18	

Primers were built using Beacon Designer 5.01 (Premier Biosoft International, http://www.premierbiosoft.com) using public gene bank sequences and are listed in Table 1. The iQ5 Optical System was used to execute all amplification processes in a total volume of 25 µL (Bio-Rad Laboratories Inc., Hercules, CA, USA). One µL cDNA, 1 µL primer (100 nM per primer), 12.5 µL 2 Power SYBR Green PCR Master Mix (Applied Biosystems, Foster City, CA, USA), and 10.5 µL RNAse/DNAse-free water were used in each well. Thermal cycling conditions were as follows: 1 cycle at 94 degrees Celsius for 10 min, 40 cycles at 95 degrees Celsius for 15 s, 56–62 degrees Celsius (annealing temperature) for 30 s, and 72 degrees Celsius for 25 s. The Pfaffle technique was used to interpret the results, and the threshold cycle (Ct) values were standardized to the expression rate of GAPDH as a housekeeping gene. All responses were carried out in triplicate, and each experiment included negative controls.

#### Pronase test

The anti-proliferative impact of pronase-treated or untreated secretions on the HT-29 cell line was explored to establish the types of chemicals that play a crucial role in the cytotoxic effects of cell-free supernatant on malignant cells, as well as possible protein-based effects. Pronase (Roche Applied Science, Penzberg, Germany) was added to the CFS at a concentration of 1 mg mL^−1^ for protein content digestion, and the mixture was incubated at 37 °C for 30 min. After deactivating pronase by heat denaturation, both treated and untreated supernatants were tested for live cells using the MTT method.

### Anti-inflammatory activity

To investigate the effect of probiotic *Enterococcus faecalis* KUMS-T48 strain on the expression of interleukin 10 (IL-10) and interleukin 1β (IL- 1β) inflammation-related genes in HT-29 cancer cell line, cells were treated with different concentrations of CFS (50, 100, 150 and 200 mcg mL^−1^) in 25 cm flasks as well as one flask as a control in RPMI-1640 culture medium. After 48 h, the cells were separated from the bottom of the flask.

RNA extraction was done using high pure kits of Roche Company based on the company's protocol, and then the concentration of extracted RNA was determined using a nanodrop spectrophotometer. Then, due to the instability and single-stranded nature of RNA, cDNA synthesis was performed using a Fermentase kit, and Real-Time PCR technique with primers listed in Table 1 were used to investigate changes in the expression of IL-10 and IL-1β genes as effective genes in inflammation.

### Statistical analysis

All data were analyzed using one-way ANOVA and Duncan statistical tests using SPSS 19.0 software. A p-value (*P < 0.05; **P < *0.01*) was considered statistically significant. Data were presented as the mean ± standard deviation of three measurements.

## Results

### Antimicrobial activity

The CFS of *E. faecalis* KUMS-T48 showed significant anti-pathogenic activities against indicator microorganisms. This strain showed strong effects on *B. subtilis* and *L. monocytogenes* and a moderate effect on *Y. enterocolitica*, while indicating a weak effect on *K. pneumoniae* and *E. coli*. Overall, *E. faecalis* KUMS-T48 inhibited the growth of Gram-positive indicator bacteria more than Gram-negative indicator bacteria (Table 2). Also, neutralizing the cell-free supernatant and treating it with the catalase enzyme reduced the antibacterial effects of *E. faecalis* KUMS-T48 by removing the effects of acid and hydrogen peroxide, while the antimicrobial effects of the cell-free supernatant were completely eliminated after the filtrates were subjected to the proteinase K enzyme.

**Table 2:** Antibacterial effects of different types of *Enterococcus faecalis* KUMS-T48 cell-free supernatant.

Experiments	Indicator bacteria				
	*Listeria monocytogenes* (PTCC 1163)	*Escherichia coli* (PTCC 1276)	*Klebsiella pneumoniae* (ATCC 43,816)	*Yersinia enterocolitica* (ATCC 2715)	*Bacillus subtilis* (ATCC 19,652)
Crude filtrate (Acidic)	S (27 mm)	W (8 mm)	W (6 mm)	M (14 mm)	S (24 mm)
Filtrate treated with NaOH (Neutral)	S (23 mm)	- (0 mm)	- (0 mm)	M (12 mm)	M (16 mm)
Filtrate treated with catalase	S (27 mm)	W (5 mm)	W (4 mm)	W (7 mm)	S (22 mm)
Filtrate treated with proteinase K	– (0 mm)	– (0 mm)	– (0 mm)	– (0 mm)	– (0 mm)

Anti-pathogenic activity was divided into strong (S: diameter ≥ 20 mm), moderate (M: 10 mm < diameter < 20 mm), weak (W: diameter ≤ 10 mm), and (–: without inhibition zone).

### Anti-proliferative activity

#### Cell viability assessment by MTT test

**Figure 1:**
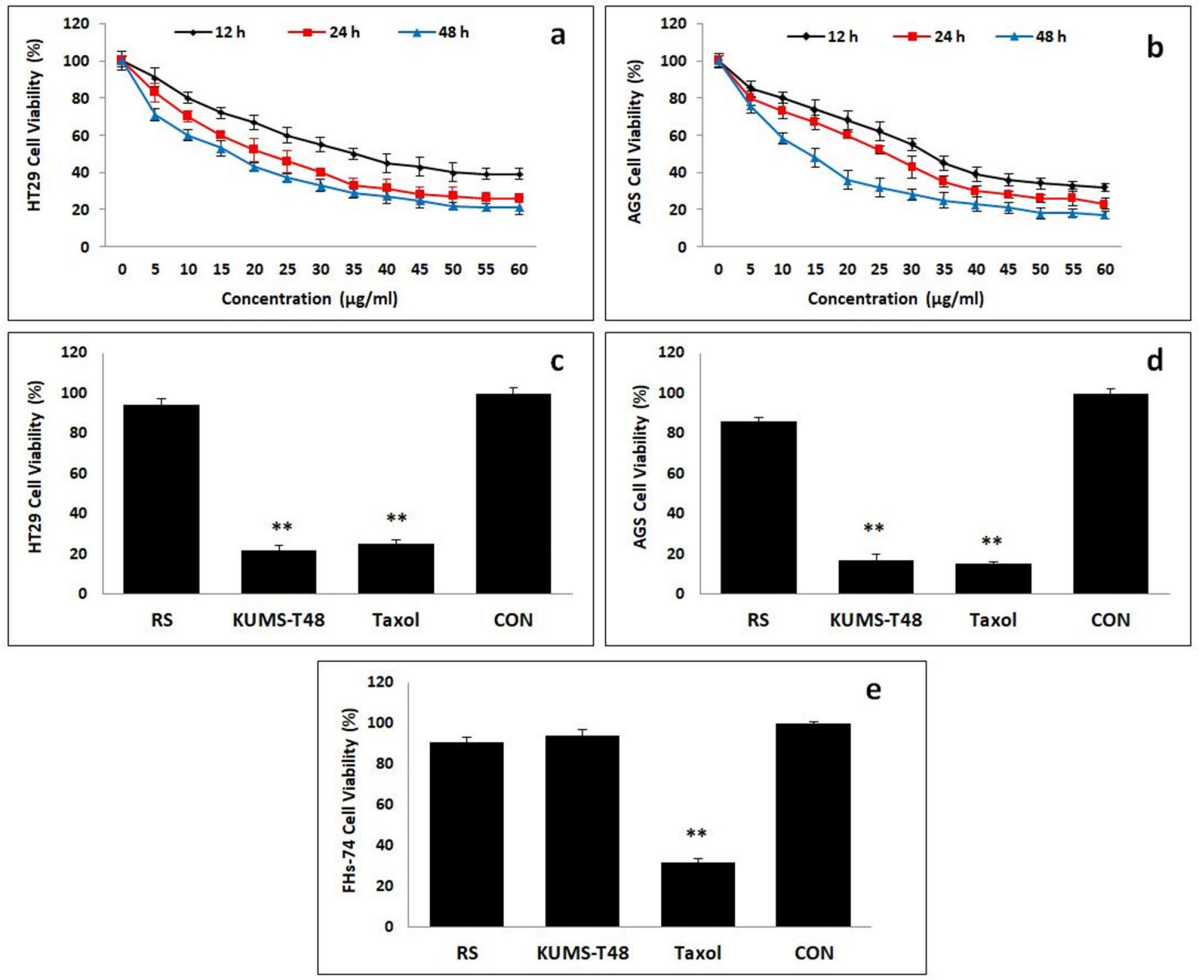
The cytotoxic effects of *E. faecalis* KUMS-T48 cell-free supernatant on cancer cell lines. Asterisks illustrate the significant differences (**P < 0.01). Error bares represent standard deviation of each mean. The effect of different concentrations of CFS on the cell viability of HT-29 (**a**) and AGS (**b**) at three time points 12, 24, and 48 h. The effects of 50 µg mL^−1^ of CFS on cancerous and normal cell lines for 48 h: (**c**) HT-29 cells, (**d**) AGS cells, and (**e**) FHs-74 cells. KUMS-T48: CFS of *E. faecalis* KUMS-T48, CON: Untreated cancer cell line, RS: reference strain (*E. faecalis* PTCC 1774) for comparison, and Taxol: positive control.

The anti-proliferative effects of *E. faecalis* KUMS-T48 secretions were time- and dose-dependent, revealing that the cell viability of both treated cancer cell lines was gradually decreased by increasing incubation time and applied dose, with the greatest anti-proliferative effect observed in the last three highest doses (50–60 g mL^−1^) after 48 h. The cytotoxic time and dose-dependent curves were used to calculate the 50% inhibitory concentration (IC_50_) of strain cell-free supernatant as an indication of anti-proliferative activity. After 12 h of incubation, the IC_50_ values of *E. faecalis* KUMS-T48 secretions on HT-29 and AGS cancer cell lines were respectively 39 and 32 µg mL^−1^, which reached 21 and 17 µg mL^−1^ at the end of the incubation period (48 h), respectively (Fig. 1, panels a,b).

The cytotoxic effects of *E. faecalis* KUMS-T48 secretions (50 µg mL^−1^) on AGS, HT-29, and FHs-74 cell lines for 48 h have been illustrated in Fig. 1, panels c–e. Compared with the untreated cancer cell line and reference strain (*E. faecalis* PTCC 1774) as control, cell viability decreased significantly (*P* < 0.01) in both human cancer cell lines treated by *E. faecalis* KUMS-T48 cell-free supernatant, similar to the effect of Taxol, indicating that the cell-free supernatant had the same anticancer effect (Fig. 1, panels c,d). Furthermore, while the percentage of cell viability of Taxol-treated FHs-74 cells decreased significantly to 31% during the incubation time (*P* < 0.01), the cell viability of these cells treated with *E. faecalis* KUMS-T48 cell-free supernatant was found to be 94% at the same time (Fig. 1, panel e), compared to the untreated cells and the control group (treated with *E. faecalis* PTCC 1774).

#### Apoptosis assessment by DAPI staining

**Figure 2:**
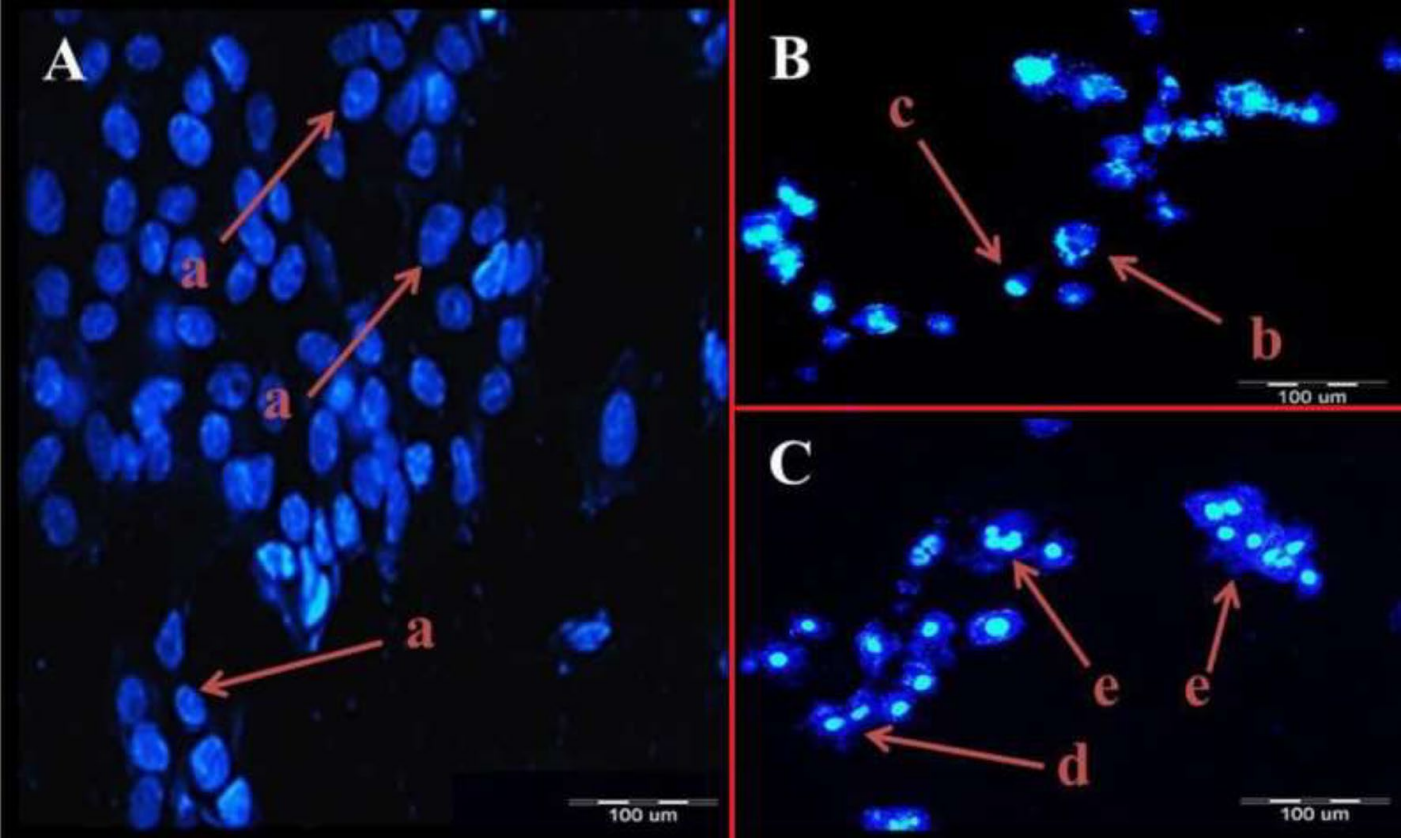
Fluorescent photomicrographs of DAPI-stained AGS cancer cell line after treatment with *E. faecalis* KUMS-T48 cell-free supernatant (50 µg mL^−1^). Panels represent; (**A**) Untreated AGS cell line, (**B**) treated AGS cell line after 24 h incubation, (**C**) treated AGS cell line after 48 h incubation: a: blue intact normal cell, b: membrane blebbing, c: cell shrinkage, d: nucleus fragmentation, and e: apoptotic bodies.

The results revealed that the numbers of apoptotic cells with the fragmented and condensed nucleus were significantly higher than normal cells (*P* < 0.05) in both cancer cell lines after treatment with *E. faecalis* KUMS-T48 cell-free supernatant (50 µg mL^−1^) for 24 h. The fluorescent photomicrographs of DAPI-stained AGS cancer cells are shown in Fig. 2. As depicted in panel 2-B and panel 2-C, the treated AGS cell lines showed apoptosis symptoms, such as membrane blebbing (b), cell shrinkage (c), nucleus fragmentation (d), and apoptotic body formation (e). Meanwhile, cell shrinkage (early apoptosis), and apoptotic bodies (late apoptosis) were the predominant apoptosis signals. Also, none of the distinctive apoptotic features were observed in untreated cells (panel 2-A).

#### RNA analysis of apoptotic genes

**Figure 3:**
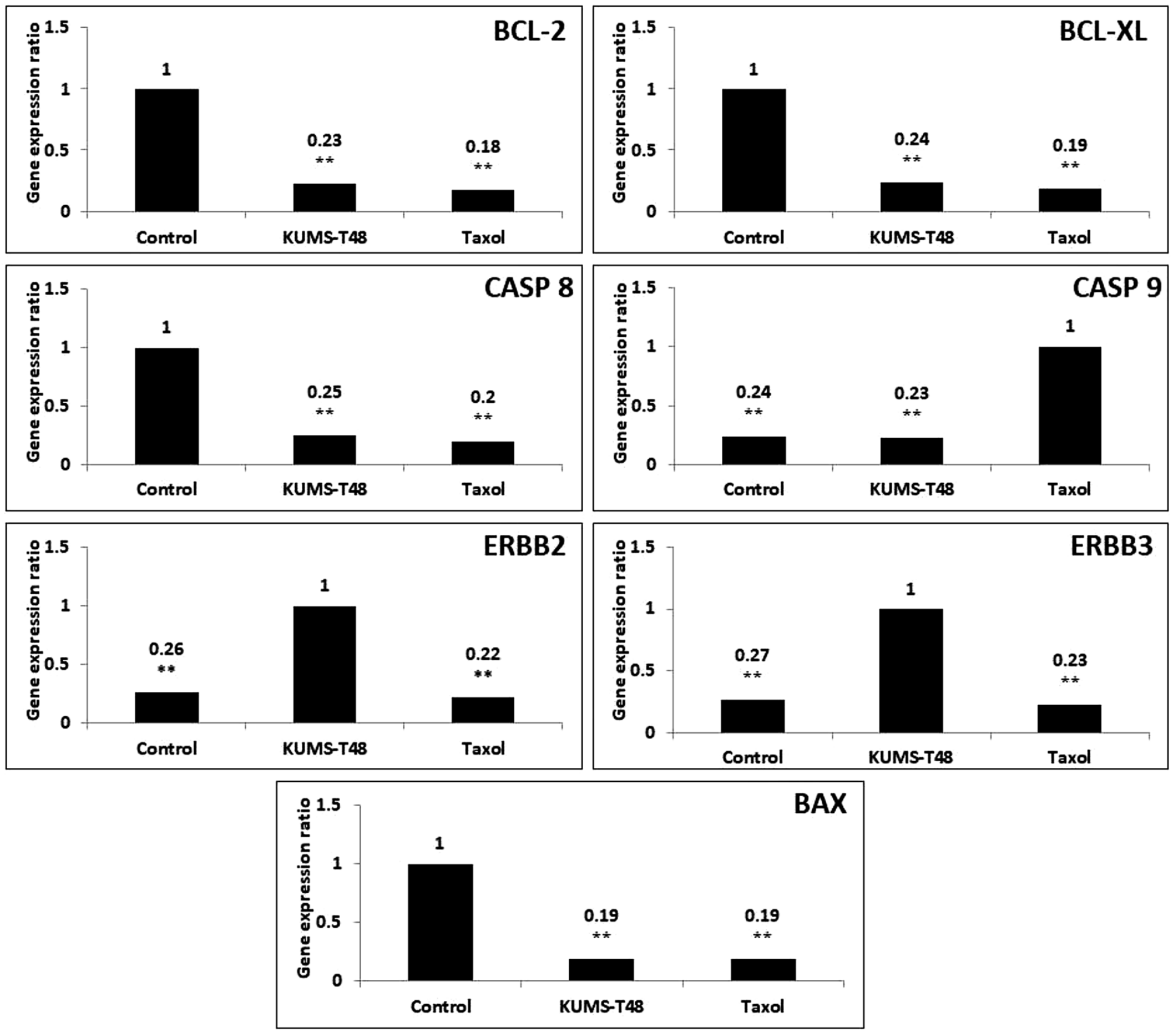
The apoptosis related genes expression ratio in AGS cancer cells treated by 50 µg mL^−1^ of *E. faecalis* KUMS-T48 cell-free supernatant and Taxol for 24 h. Asterisks illustrate the significant differences (**P < 0.01).

For this, seven apoptotic expression genes including ErbB-2, ErbB-3, BAX, BCL-2, BCL-XL, CASP-8, and CASP-9 were analyzed by Real-Time PCR. The down-regulation in BCL-2, BCL-XL, BAX, and CASP-8 genes by *E. faecalis* KUMS-T48 cell-free supernatant was similar to Taxol, but the expression of CASP-9 (starter gene in intrinsic apoptosis pathway) and ErbB-2, ErbB-3 (anti-apoptotic genes) was significantly different in *E. faecalis* KUMS-T48 and Taxol treated groups (Fig. 3).

#### Pronase test

**Figure 4:**
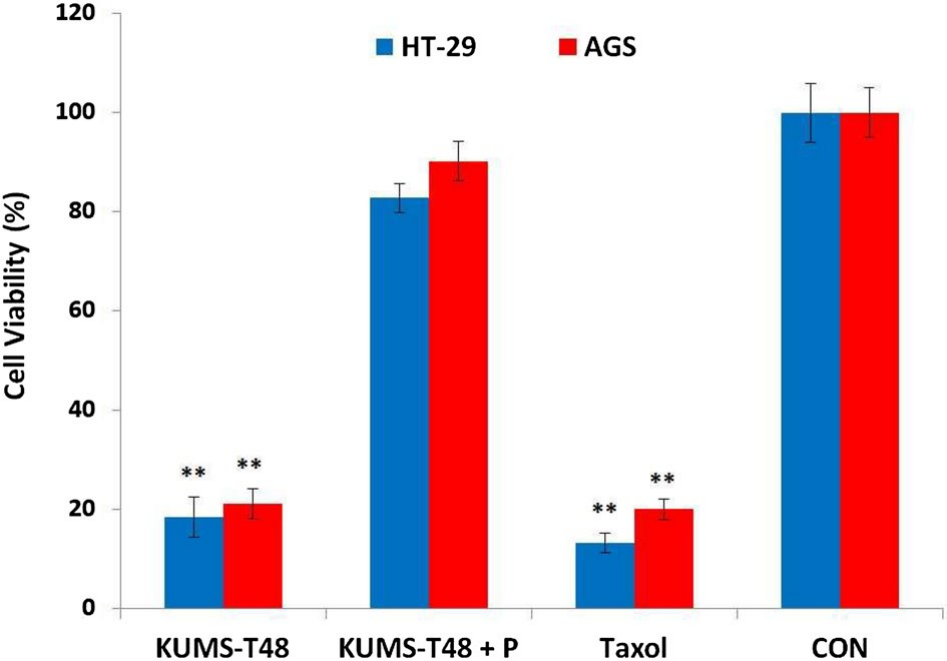
The cytotoxic effects of *E. faecalis* KUMS-T48 and its pronase treated secretions (50 µg mL^−1^) on the AGS and HT-29 cancer cell lines for 48 h. CON: Untreated cancer cell line, P: pronase treated cell-free supernatant, and Taxol: positive control. Asterisks illustrate the significant differences (**P < 0.01). Error bares represent standard deviation of each mean.

The pronase-untreated secretions showed significant cytotoxic effects (*P* < 0.01) on both HT-29 and AGS cell lines same as Taxol, compared to control sample. The cell viability percentage of treated cells with *E. faecalis* KUMS-T48 cell-free supernatant and Taxol during incubation time (48 h) were respectively detected 18% and 13% for HT-29 and 21% and 20% for AGS cell lines. However, compared to those on un-treated cells, secretions had very little effect on cancer cells after treatment with pronase, highlighting the critical role of secreted proteins in cytotoxic effects (Fig. 4).

### Anti-inflammatory effects

**Figure 5:**
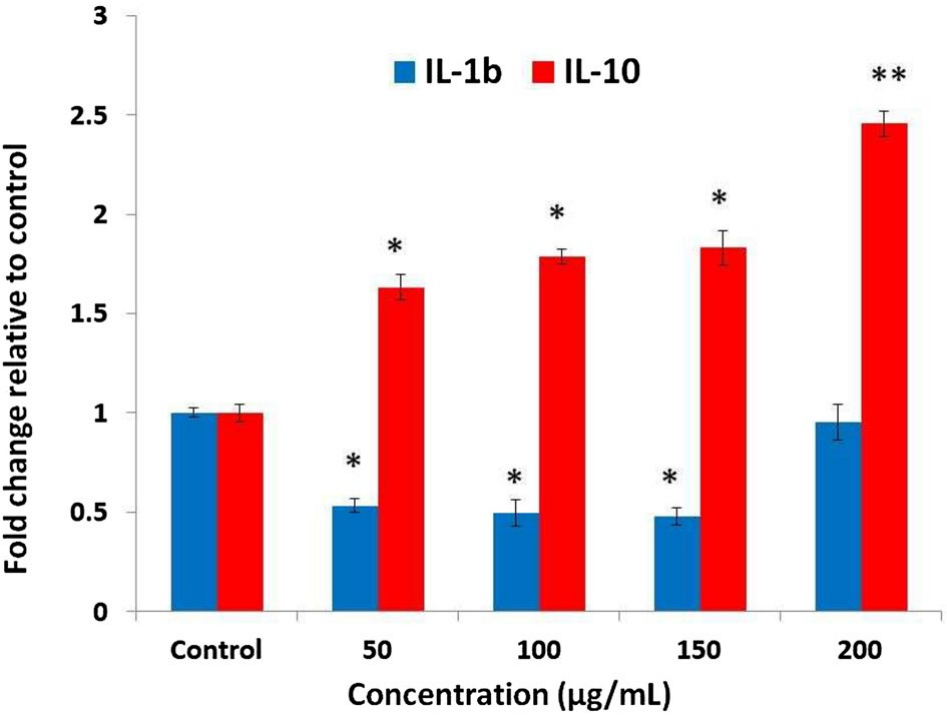
The changes of interleukin 10 and interleukin 1β genes expression in HT-29 cancer cell line after 48 h treatment with different concentration of *E. faecalis* KUMS-T48 cell-free supernatant. Asterisks illustrate the significant differences (*P < 0.05 and **P < 0.01). Error bares represent standard deviation of each mean.

In this research, the changes in interleukin 10 and interleukin 1β genes expression in cancerous cell lines was investigated. The results of this assay on cancerous cells treated with the CFS of probiotic *E. faecalis* KUMS-T48 are shown in Fig. 5. As can be seen, the expression of IL-1β inflammation-promoting gene in the HT-29 cell line was significantly decreased (*P* < 0.05) up to a concentration of 150 µg mL^−1^ in a dose-dependent manner. Conversely, the expression of the anti-inflammatory IL-10 gene treated with *E. faecalis* KUMS-T48 increased significantly with increasing the concentration of supernatant up to 200 µg mL^−1^ (*P* < 0.01).

## Discussion

In this work, traditional dairy products in the west of Iran were preliminarily screened because different preparation and processing methods can be possibly used as valuable tools to introduce novel and promising probiotic bacteria^27,28^. The findings showed that the antagonistic activity of *E. faecalis* KUMS-T48 cell-free supernatant was retained after neutralizing CFS and treatment with catalase enzyme, while its activity was completely lost by subjecting it to proteinase K enzyme. So it can be concluded that the antagonistic effect may not be related to acid or H_2_O_2_ production and could be attributed to bacteriocins. These findings are consistent with the results reported by others^29^. Similar to our results, the antagonistic activities of various *Enterococcus* strains against the diversity of pathogenic bacteria have been previously reported^1,30^. The effects of *E. faecalis* KUMS-T48 cell-free supernatant on Gram-positive bacteria were significantly higher than that of Gram-negative bacteria, which may be related to the outer membrane of Gram-negative bacteria^31^.

Probiotics, as health-promoting microorganisms, exhibit different therapeutic activities. However, anti-carcinogenic activity is the most interesting property that has been linked to probiotics^32,33^. Most studies on the role of probiotic bacteria in cancer prevention have focused on their anti-colorectal cancer effects^34,35^ whereas, our results indicate that the cell-free supernatant of *E. faecalis* KUMS-T48 displayed cytotoxic effects on other cancer cell lines, similar to Taxol. Compared to other cancer cells such as hematological cell lines, HT-29 and AGS (cell lines of epithelial origin), are less sensitive to cytotoxic agents and drugs^36^. Therefore, the effective agents on them may have better conditions to be used as anticancer agents.

As a conventional anticancer drug, Taxol is relatively expensive, and its original source (Pacific yew tree) is limited. In addition, this drug has a cytotoxic effect on non-cancerous cells^37^. Hence, finding safe and inexpensive alternatives such as probiotic metabolites is important. Anti-cancer agents can generally show cytotoxic effects on some sensitive cells and tissues, such as the bone marrow, stem cells, and hair follicle cells^38,39^. In this study, *E. faecalis* KUMS-T48 cell-free supernatant did not show any significant cytotoxic effects on rapidly dividing normal cell lines (FHs-74), so they could be prescribed more safely.

In the present study, the anti-proliferative effect of pronase-treated/untreated secretions was investigated on the cell lines. Our results show that active proteins serve a key function in the cytotoxicity of the cell-free supernatant. Proteins suppress the cancer cell lines by binding to pro-carcinogenic compounds, carcinogenic fecal enzymes, or mutagenic substances^40^. This process explains the cytotoxic effects of pronase-untreated secretions of *E. faecalis* KUMS-T48 on the AGS cancer cell line.

The association between the anticancer activity of therapeutic agents and apoptosis has been shown in different cell lines, such as human cervical^41^, prostate^42^ or colon^20^ cancer cell lines. Methods utilized to assess apoptosis ranged from classical biochemistry and simple light microscopy to the application of sophisticated instruments, such as flow cytometer^43^. However, cellular morphological characteristics should be considered when determining the mode of cell death. Therefore, the DAPI staining method flowing fluorescent microscopy is still considered the gold standard for analyzing apoptosis^44^. Various forms of apoptotic bodies were observed in cancer cells treated with *E. faecalis* KUMS-T48 cell-free supernatant. These changes in cell morphology during apoptosis have also been reported by other researchers^45^. The apoptotic cells can be distinguished from necrotic and viable cells based on nuclear morphology, such as nucleus fragmentation and chromatin condensation^46^. The morphological apoptotic results of this work indicate that apoptosis is the main cytotoxic mechanism for the cell-free supernatant of *E. faecalis* KUMS-T48 on cancer cell lines and the necrotic bodies were rarely observed.

The expression of pro-apoptotic markers (CASP and BAX) and anti-apoptotic proteins (BCL-2 and BCL-XL) is used as an indicator of apoptosis induction in tumor cells^47^. We also showed that the expression of intrinsic apoptosis blocker genes (BCL-2 and BCL-XL), CASP-8 gene (starter gene in TNF-α apoptosis pathway), and BAX (crucial gene in extrinsic IL-3 mediated apoptosis pathway) were significantly down-regulated by *E. faecalis* KUMS-T48 compared to the untreated control group. The cell-free supernatant from *E. faecalis* KUMS-T48 increased the expression of ErbB-2 and ErbB-3 genes, while Taxol increased the expression of CASP-9, suggesting alternative apoptosis-inducing mechanisms. According to these results, the activation of apoptosis by *E. faecalis* KUMS-T48 The cell-free supernatant from *E. faecalis* KUMS-T48 increased the expression of ErbB-2 and ErbB-3 genes, while Taxol increased the expression of CASP-9, suggesting alternative apoptosis-inducing mechanisms is associated with the down-regulation of anti-apoptotic genes (BCL-2 and BCL-XL), but differs from Taxol's apoptosis induction (intrinsic mitochondrial apoptosis) route. The molecular mechanisms of the pro-apoptotic effects of human-derived *Lactobacillus reuteri* (ATCC 6475) on myeloid leukemia-derived cells have previously been investigated, with findings indicating down-regulation of nuclear factor kappa B (NF-kappa B)-dependent gene products that mediate cell survival-related genes (BCL-2 and BCL-XL). *Lactobacillus paracasei* M5L, according to Hu et al.^48^, may trigger apoptosis in HT-29 cells through reactive oxygen species production, followed by CRT-accompanied endoplasmic reticulum stress and S phase arrest. The impact of probiotic *Bacillus polyfermenticus* on the proliferation of human colon cancer cells such as HT-29, DLD-1, and Caco-2 cells has been discovered. It has been observed that the apoptosis-inducing effect of *Bacillus polyfermenticus* metabolite is connected to the repression of the ErbB-2 and ErbB-3 genes, which resulted in this strain's anti-tumor characteristics^49^. Furthermore, the anticancer effects of cell-bound exopolysaccharides (cb-EPS) isolated from *Lactobacillus acidophilus* 606 on HT-29 colon cancer cells have been shown by the expression of BAX gene^50^.

Previous studies implies that various bacteria may have potential to be used as anti-inflammatory agents^51^. Probiotic lactobacilli, bifidobacteria, and *E. faecium* have been linked to anti-inflammatory effects in the past^52,53^. In the present study, the cell-free supernatant from *E. faecalis* KUMS-T48 increased the expression of ErbB-2 and ErbB-3 genes, while Taxol increased the expression of CASP-9, suggesting alternative apoptosis-inducing mechanisms of probiotic *E. faecalis* KUMS-T48 demonstrated a strong anti-inflammatory impact, as evidenced by a decrease in interleukin 1β gene expression and an increase in the anti-inflammatory interleukin-10 gene expression in the HT-29 cell line. Reducing the release of inflammatory mediators such as IL-1β, or increasing the level of IL-10 as an anti-inflammatory cytokine, are considered in studies investigating anti-inflammatory effects^54^. IL-1β is involved in inflammation and tumor growth by stimulating angiogenesis in the cancerous cells^55^. However, IL-10 is a strong inhibitor of the synthesis of pro-inflammatory cytokines such as TNF-α^56^.

In conclusion, the cytotoxic effects of *E. faecalis* KUMS-T48 secretions on cancerous HT-29 and AGS cell lines were similar to that of Taxol, which is a conventional anticancer drug. Taxol possesses high cytotoxicity on normal cell lines, but the cell-free supernatant from *E. faecalis* KUMS-T48 increased the expression of ErbB-2 and ErbB-3 genes, while Taxol increased the expression of CASP-9, suggesting alternative apoptosis-inducing mechanisms of *E. faecalis* KUMS-T48 did not show any significant cytotoxic effects on rapidly dividing FHs-74 normal cell lines. Furthermore, *E. faecalis* KUMS-T48 cell-free supernatant showed remarkable antibacterial activity and anti-inflammatory effects. These antimicrobial properties or anti-proliferative effects of the cell-free supernatant that were induced by apoptosis were lost after subjection to proteolytic enzymes. Hence, these secreted proteins have the potential to be introduced as anticancer therapeutics. Of course, they should be comprehensively examined in terms of physicochemical, structural, and functional properties before use.

## Data Availability

The datasets generated and/or analysed during the current study are available in the NCBI GeneBank repository, Accession Number MW433678.
